# Choice of Healing Agent for Self-Healing Asphalt Concrete

**DOI:** 10.3390/ma16247542

**Published:** 2023-12-07

**Authors:** Sergei Sergeevich Inozemtcev, Evgeniy Valerievich Korolev, Trong Toan Do

**Affiliations:** 1 Department of Building Materials Science, National Research Moscow State University of Civil Engineering, 129337 Moscow, Russia; 2 Department of Construction Materials Technology and Metrology, St. Petersburg State University of Architecture and Civil Engineering, 190005 St. Petersburg, Russia; korolev@nocnt.ru; 3 Department of Building Materials, Hanoi Architectural University, Hanoi 12109, Vietnam; trongtoan007@gmail.com

**Keywords:** self-healing, healing agent, asphalt concrete, AR-polymer, sunflower oil, solubility

## Abstract

The choice of a substance as a healing agent for asphalt concrete is determined by the scientific experience of researchers and the results of exploratory studies. There are no standard approaches for selecting healing agents or assessing their compatibility with the matrix components in asphalt concrete. However, such methods would make it possible to systematize research in the field of self-healing asphalt concrete and significantly expand the list of healing agents potentially suitable for encapsulation and ensuring the formation of a healing effect. An approach has been proposed for studying a substance and assessing the suitability of its use as a healing agent during encapsulation, using alginate technology in terms of solubility, homogeneity in a system with bitumen, and adhesive strength. This set of indicators can be used in the development and design of self-healing asphalt concrete, as well as for expanding the list of healing agents that can be used to implement self-healing technology. This article discusses sunflower oil and AR polymers as healing agents for self-healing asphalt concretes. The substances under consideration are capable of forming a homogeneous system ∆*δ* → 0 with bitumen, and the double systems “SfO-bitumen” and “ARP-bitumen” have a Gibbs energy value ∆G < 0, which confirms this. The studied healing agents are able to form an emulsion in alginate aqueous solutions, which was confirmed by the structuring effect and the extreme influence of their concentration on viscosity. The strength of the adhesive bonds under the influence of SfO was 14.2% of the initial value of the tensile strength during splitting. Under the influence of ARP, the strength of the adhesive bonds was 5.8% of the initial value of the tensile strength at splitting. The use of an activator in asphalt concrete makes it possible to increase the strength of the adhesive bonds to 25–45% of the initial splitting strength.

## 1. Introduction

The development of building materials for various functional purposes or their modification involves the use of new technological modes and components to solve the assigned problems [1,2,3,4]. One of the stages of this process is to assess the compatibility of new modes and components with existing technology for the production of building materials. This problem is actualized by the task of developing materials with specific properties, such as the ability to self-heal [5,6,7,8].

During the process of developing building materials with a self-healing function, it is necessary to take into account both the parameters of the material structure and its production technology. The implementation of self-healing in asphalt concrete is complicated by the specific conditions of the production process and the structural features of the thermoplastic matrix, as well as the operational and climatic conditions [9,10,11].

**Figure 1:**
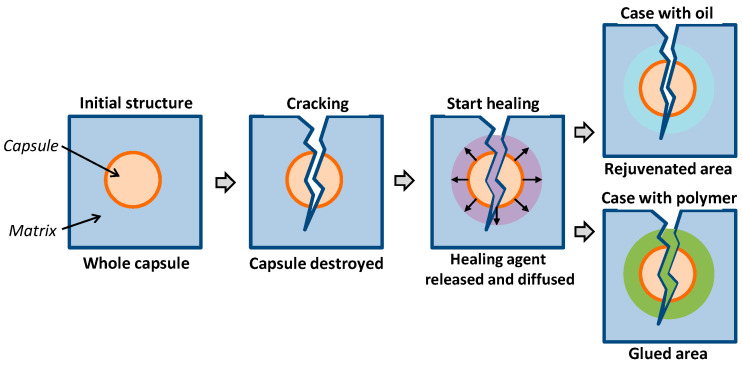
Scheme of the self-healing process with various healing agents.

The implementation of the self-healing function in asphalt concrete, in most cases, is carried out using encapsulated healing agents [12,13,14,15,16,17,18,19]. Researchers have proposed using various types of hydrocarbon compounds as a healing agent: vegetable oils [12,13,14,15,16], industrial rejuvenators [17], and their mixtures with polymers [18] or pure polymers [19]. In these cases, the healing effect in asphalt concrete can be achieved by the spontaneous “entanglement” of molecules of organic compounds in the zone of bond rupture that occurred during the formation of a defect or by the gluing of the defect surfaces through the formation of new adhesive bonds during the structural formation of the modifier (Figure 1).

The process of spontaneous entanglement of molecules depends on the proximity of the molecules to each other and the speed of spontaneous movement, which increases with increasing temperature. Thus, the kinetic energy of molecules and the average distance between them increase as the temperature increases. This leads to natural physical phenomena: an increase in the volume of the material and a decrease in the viscosity of the thermoplastic material [20]. Spontaneous fusion will occur in the defect (crack) zone due to a decrease in surface tension at the “material–air” interface if its surfaces are sufficiently close.

The formation of new adhesive bonds is a two-stage process. In the first stage, the healing agent (adhesive) moves into the material (substrate) with a defined orientation of molecules in the boundary layer, and in the second stage, the adhesive and substrate interact with the participation of van der Waals and/or other forces during the formation of chemical bonds. Covalent bonds act at a distance between molecules of less than 0.5 nm, while ionic bonds and van der Waals forces act at a distance from 1 to 100 nm [21,22].

Often the choice of a substance as a healing agent for asphalt concrete is determined by the scientific experience of researchers and the results of exploratory studies. There are no standard approaches for selecting healing agents and assessing their compatibility with the matrix component in asphalt concrete. However, such methods would make it possible to systematize research in the field of self-healing asphalt concrete and significantly expand the list of healing agents potentially suitable for encapsulation and ensuring the formation of a healing effect.

The effectiveness of a healing agent in asphalt concrete depends not only on its chemical nature but also on the degree of its compatibility with the bitumen binder. The more soluble the components of the system are in each other, the more intense the process of interaction between them and the greater the effect achieved when decapsulating the healing agent. In practice, determining the compatibility of substances requires significant material and time costs, and therefore theoretical methods for determining solubility have obvious advantages.

There are various calculation methods that allow you to determine the compatibility of substances in mixtures of two components. A prerequisite for the formation of a thermodynamically stable mixture is a decrease in the free energy of the system Δ*G* when mixing the components [23]:

(1) ∆G=∆H−T∆S<0,

where Δ*H* is the heat of mixing of the components, Δ*S* is the entropy of mixing of the components, and *T* is temperature.

A feature of systems containing high-molecular organic compounds is the great influence of the entropy factor. In accordance with the Flory–Huggins theory, the change in entropy when mixing components can be determined by the equation:

(2) $$∆S=-R\sum_{i}^{n}x_{i}ln\Phi_{i},$$

where *R* is the universal gas constant, *x_i_* is the number of moles of the *i*-th component, Φ*_i_* is the volume fraction of the *i*-th component, and *n* is the number of components in the mixture.

The heat of mixing of the components of a binary mixture Δ*H_m_*, provided there are no specific interactions (solvation, complexation, etc.), according to the theory of regular solutions, can be determined using the Hildebrand equation [24]:

(3) $${∆H}_{m}=V_{c}{\left(\delta_{1}-\delta_{2}\right)}^{2}\Phi_{1}\Phi_{2},$$

where *V_c_* is the volume of the mixture and δ*_i_* is the solubility parameter of the *i*-th component. 

When mixing mutually soluble substances, the volume of the mixture cannot be calculated as an algebraic sum of the absolute volumes of the components. In this case, the volume of the mixture is calculated using the Gibbs–Duhem equation:

(4) $$V_{c}=\sum_{i=1}^{n}x_{i}v_{i},$$

where *v_i_* is the molar volume of the *i*-th component.

In the literature, there are several computational and experimental methods for determining the solubility parameter. However, researchers do not have a consensus on the issue of determining the values of δ*_i_*. The solubility parameter values for one substance determined by different methods vary greatly (Table 1).

Each presented technique has certain advantages. For example, variable calculations carried out according to Formula (5) indicate that with an increase in temperature for every 10 °C, the value of the solubility parameter of a substance decreases by 0.1–0.2%. Therefore, even with significant changes in temperature (up to 100 °C), the error in calculations, which do not take into account the direct effects of temperature on changes in the solubility parameter, is minimal.

The use of a semi-empirical method (Formula (6)) involves an experimental determination of the surface tension of a substance, which is associated with certain difficulties, especially that of determining *σ_i_* at elevated temperatures (for example, for thermoplastic melts).

The calculated values using the Small method (Formula (7)) are close to the experimental data. However, it is unknown how accurately this method reflects the solubility parameter values of low molecular weight substances, although the physical justification of the method is applicable for both types of substances.

By definition, the intensity of an intermolecular interaction is equal to the potential energy of the interaction between molecules per unit volume of a substance and, in the case of a liquid approaching the boiling point, is equivalent to the work of removing interacting molecules over an infinitely large distance, which practically corresponds to the evaporation of a substance. If the attraction of molecules is due to van der Waals forces, then the solubility parameter is calculated using the formula:

(8) $$\delta_{i}=\sqrt{\frac{E}{V_{m}}},$$

where *E* is the cohesion energy and *V_m_* is the molar volume.

An analysis of the results of calculating the values of the solubility parameter of some organic substances [28] showed that the values of the solubility parameter calculated using Equations (5)–(8) differ. Values of the solubility parameter that are close in absolute value are obtained when using Formulas (5) and (8), as well as Formulas (6) and (7). This indicates that two different approaches were used to determine the solubility parameter. Equations (6) and (7) reflect intermolecular interactions in condensed systems, provided that the temperature of the system is not close to the boiling point of the solvent. Equations (5) and (8) take into account the energy required to remove a molecule of a substance from the volume of the system to the distance where the action of van der Waals forces ceases. Therefore, the values of the solubility parameter of the same substance calculated using these two approaches differ. Since when considering condensed systems at temperatures not close to the boiling point of the solvent, it is not necessary to take into account the evaporation energy of substances, it is advisable to use the approach reflected by Equations (6) and (7). Of the two proposed methods for this approach, Small’s method is more acceptable, because in contrast to the use of Formula (6), it allows the use of calculated data without resorting to experiments.

Two healing agents (HA) were investigated in this work. As agents for self-healing, conventional sunflower oil (SfO) and a thiol-containing urethane AR-polymer (ARP) were used. Self-healing due to matrix rejuvenation can be realized using sunflower oil and self-healing due to defect gluing can be implemented using AR polymers.

## 2. Materials and Methods

**Figure 2:**
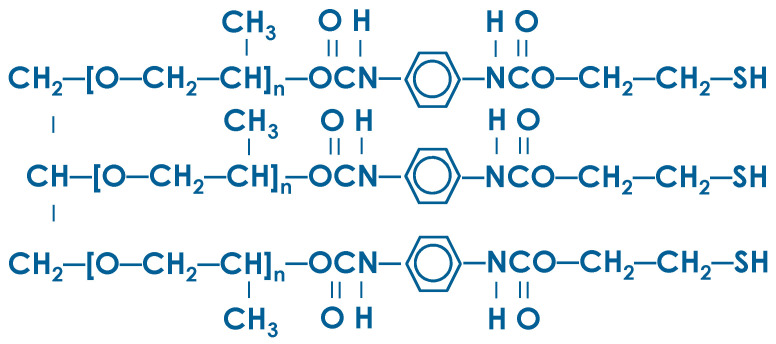
Structural formula of AR-polymer.

AR-polymer is a thiol-containing urethane polymer with terminal mercaptan groups (SH-), produced by «PolyMix Kazan» LLC (Kazan, Russia) by TU 2226-001-90014974-11 (Figure 2). AR polymer is a mixture of «» polymer (70%) and dibutyl phthalate (30%) as a plasticizer. Polymer “Aprol 3003” is a simple polyester, a product of alcoholate polymerization of propylene oxide with glycerin, which is traditionally used in the production of elastic polyurethane foams.

Polyethers (polyether polyols) are oligomeric products with a molar mass of up to 20,000 with OH-functional terminal groups. Polyethers are heterochain oligomers containing regularly repeating C-O-C ether groups in the main chain. Industrially, they are produced by the catalytic addition of cyclic ether alkylene oxides to an initiator (also often called a starter). Polyethers are highly soluble in most organic solvents (alcohols, ethers, acetone, carbon tetrachloride, etc.). The solubility in water is not the same for different brands of polyols; their solubility increases with an increasing proportion of ethylene oxide in the polyester. The solubility of polyesters in water decreases with increasing temperature. The main properties of the AR-polymer are presented in Table 2.

The main properties of sunflower oil are presented in Table 3.

Samples of water emulsions with different contents of sodium alginate (SA) from 1.8 to 3.33% and healing agent from 0 to 15% were made to study the influence of prescription factors on the dynamic viscosity of alginate emulsions.

Alginate emulsions were obtained by mixing SA and HA in water in various proportions and mixing for 2 min using an overhead stirrer with a drive rotation speed of at least 2000 rpm [33].

**Figure 3:**
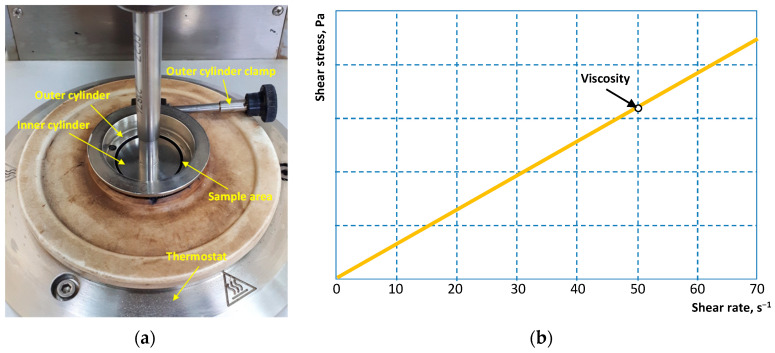
Coaxial cylinder measuring system (**a**) and viscosity determination scheme (**b**)**.**

The dynamic viscosity of the emulsions were determined using a MCR 101, (Anton Paar GmnH, Graz, Austria) rotational viscometer using a coaxial cylinder measuring system (Figure 3). The outer cylinder was filled with an emulsion sample and the inner cylinder was lowered into the sample using an automatic drive. The sample was maintained at 25 °C for 30 min before measurement. After that, the dynamic viscosity was determined at a constant shear rate of 50 s^−1^. The viscosity value was calculated as the arithmetic average of 10 measurements under the given conditions.

The SMA-15 mixtures were prepared following the aggregate gradation presented in Table 4. The binder content was 7%, resulting in an air void content of 3%, and they were 0.3% cellulose fibers by weight. Viatop-66 was used as a stabilizing additive to prevent segregation.

BND 60/90 bitumen (State standard 22245-90 [34]) was used as a binder for SMA with its main properties summarized in Table 5.

SMA cylindrical specimens with a height and diameter of 71.4 mm were manufactured by placing the required mass of the mixture into a mold and compacting it in two stages. Vibration compaction was applied for 3 min followed by hydraulic press compaction. 

The determination of the main properties of SMA-15 were carried out according to the methods of State standard GOST 12801-98 [42] (Appendix A). SMA-15 meets the requirements of State standard GOST 31015-2002 [43].

**Figure 4:**
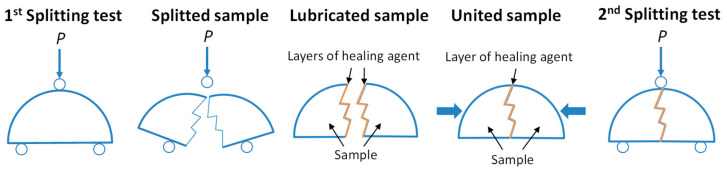
Adhesive strength test scheme.

Adhesive strength after restoration was assessed by the breaking strength of samples of SMA half-cylinders glued with a healing agent. The cylinder samples were sawn into half-cylinder samples using a circular saw. Half-cylinder samples were tested for splitting at a loading speed with a press plate of 3 mm/min after adjusting their temperature to −20 °C. The fracture surfaces of the halves of the half-cylinder samples were lubricated with a healing agent and combined with each other. After a recovery period of 3, 7, and 14 days, the samples were equilibrated to −20 °C and then re-tested for splitting. The test scheme is shown in Figure 4.

The hardener for the polymerization of the AR-polymer is an activator consisting of technical sulfur, manganese oxide (IV), and tetramethyl thiuram disulfide, mixed in a ratio of 6.1:3.7:1.0. An activator was used to increase its adhesive strength after restoration. For this purpose, SMA samples were prepared with different activator contents from 0.5 to 11.5% by weight of bitumen. 

The tensile strength during splitting was calculated using the formula:

(9) $$R_{b}=\frac{P}{2RT},$$

where *P* is the maximum bending load in N, *T* is the width of the half-cylinder sample in cm, and *R* is the radius of the half-cylinder sample in cm.

The structures of the alginate emulsions were studied using a Nikon Eclipse MA200 (Nikon Metrology Europe NV, Leuven, Belgium) optical microscope using Thixomet.Lite software (v. 3.0.0028) with calibrated electronic instruments and scales. The geometric parameters of the structures of the alginate emulsions were measured at a zoom of 200 times.

## 3. Results and Discussion

First of all, it is better to select a healing agent using technological criteria (solubility index, Gibbs energy, and rheological properties), and at the next stage by using physical-mechanical criteria (adhesive strength).

The solubility parameter is characterized by the intensity of intermolecular interaction in a substance and is equal to the energy expended in moving molecules away to a distance at which interaction forces can be neglected.

For a healing agent, the solubility parameter is equal to:

(10) $$\delta_{m}=\sqrt{\frac{E_{m}\rho_{m}}{M_{m}}},$$

where *E_m_* is the evaporation energy of the healing agent, ρ*_m_* is density of the healing agent, and *M_m_* is the molar mass of the healing agent.

For the evaporation energy of organic compounds, Trouton’s equation is applicable:

(11) $$E=kT_{b},$$

where *T_b_* is the boiling point and *k* is a constant equal to 89.12 J/(mol∙K).

The compatibility of the healing agent and bitumen in the asphalt concrete matrix is characterized by the value ∆δ =|δ*_b_* − δ*_m_*|, according to which the homogeneity of the system increases as ∆δ → 0. From here, under the condition δ*_b_* = δ*_m_*, we obtain:

(12) $$M_{m}=\frac{kT_{b}\rho_{m}}{\delta_{b}^{2}}.$$

The main properties and solubility parameters characterizing the ability to form a homogeneous system are presented in Table 6.

**Figure 5:**
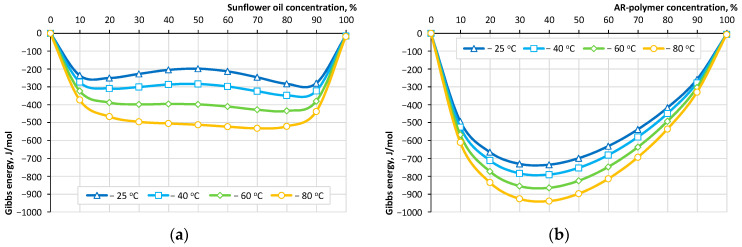
Solubility diagrams for two-component systems «sunflower oil–bitumen» (**a**) and «AR-polymer–bitumen» (**b**).

The solubility diagram of the studied healing agents in bitumen over a wide range of concentrations is shown in Figure 5.

Sunflower oil and AR polymer have a solubility index of 3.1 (J/cm^3^)^0.5^ and 2.7 (J/cm^3^)^0.5^, the values of which differ from the solubility index of bitumen by 1.2 and −0.6 (J/cm^3^)^0.5^. Thus, as ∆δ → 0, the homogeneity of the system increases, i.e., the substances in question are capable of forming a homogeneous system with bitumen. Analysis of the data’s dependence on the Gibbs energy in two-component “healing agent–bitumen” systems while varying their concentration shows that AR-polymer, proposed as an active healing agent, has the ability to form a homogeneous system. It can be seen that all binary systems have a value ∆G < 0, indicating the possibility of the formation of homogeneous systems (dissolution of the components). The “AR-polymer–bitumen” system has the largest absolute values, and the “sunflower oil–bitumen” system has the smallest, which also increase with increasing temperature. An increase in temperature ensures the formation of a homogeneous mixture, which is very important for increasing the efficiency of self-healing, as confirmed by numerous experiments [44].

**Figure 6:**
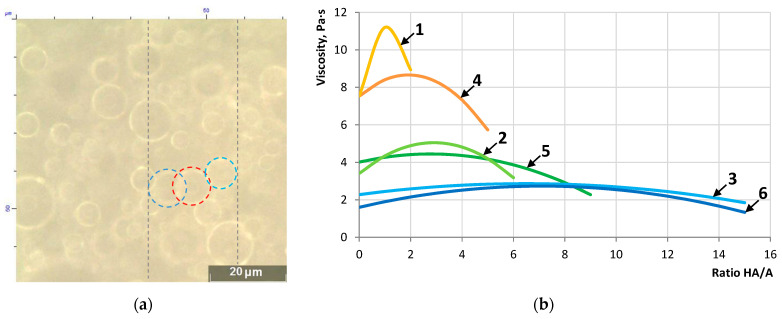
Micrograph of the emulsion (**a**) and dependence of the emulsion viscosity on content ARP with SA = 3.33% (1), 2.5% (2), 2.08% (3), and (**b**) SfO with SA = 3.0% (4), 2.3% (5), 1.8% (6).

An important condition for implementing the technology of encapsulating a healing agent using alginate technology is the possibility of creating their emulsions based on aqueous solutions of sodium alginate (Figure 6a). The results of determining the viscosity of the studied emulsions are presented in Figure 6b.

**Figure 7:**
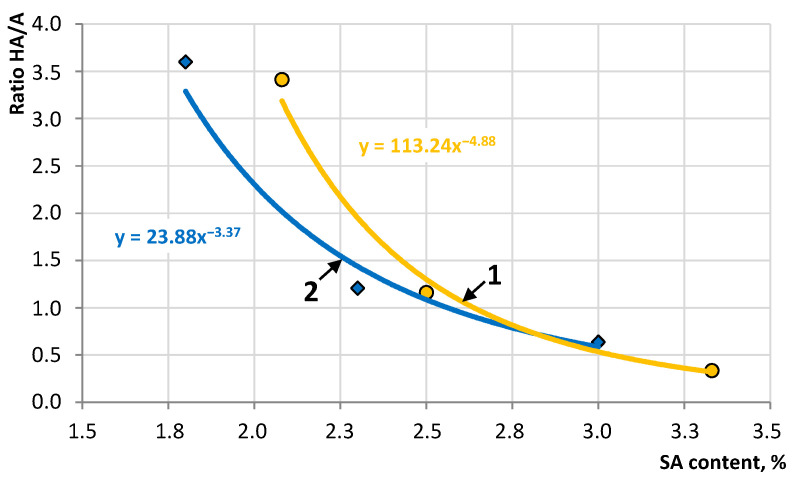
Dependence of the HA/A ratio on the content of sodium alginate for emulsions with maximum viscosity: 1—ARP and 2—SfO.

The results show that the healing agents (SfO and ARP) form an emulsion in aqueous sodium alginate solutions. The viscosity dependences are described by an equation close to the G.K. Batchelor formula [45], which indicates the structuring effect of healing agents on the emulsion. This allows us to determine the prescription limit when encapsulating healing agents (Figure 7), consistent with previously obtained data [46].

The studied healing agents are able to form an emulsion in alginate aqueous solutions, which was confirmed by the structuring effect and the extreme influence of their concentration on viscosity. A recipe limit has been established that allows you to control the technological properties of the emulsion to increase the efficiency of the encapsulation process. The formulation boundary is characterized by the dependence of the *HA/A* ratio on the SA content, at which maximum viscosity values are reached; for SfO it is described by the equation *HA*/*A* = 23.88·*SA*^−3.37^, and for ARP, *HA*/*A*=113.24·*SA*^−4.88^.

Thus, sunflower oil and AR polymer are capable of forming an emulsion in aqueous solutions of sodium alginate, which allows the technology of encapsulating the healing agent.

In order to assess the healing potential of healing agents, their ability to glue defects in samples of crushed stone-mastic asphalt concrete ShMA-15 was studied. The main properties of SMA-15 are presented in Table 7.

**Figure 8:**
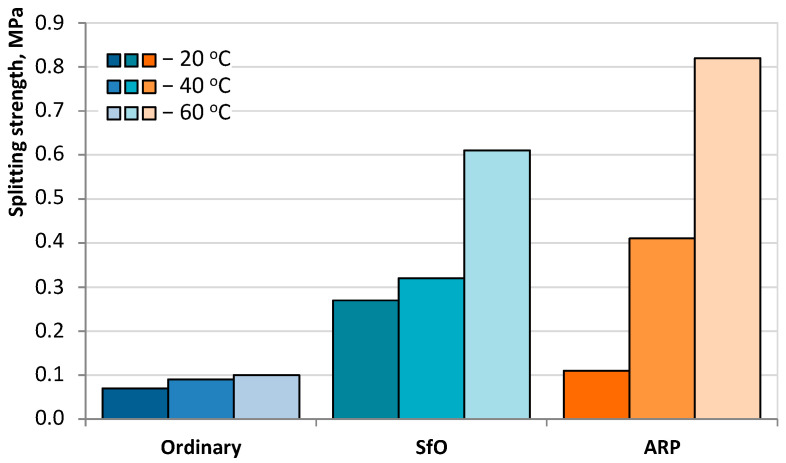
Splitting strength of the SMA samples after gluing and a rest period.

The study of the healing potential of healing agents was assessed by the change in the tensile strength during the splitting of glued half-cylinder samples. The change in ultimate strength during the splitting of control samples—half-cylinders SMA-15 glued with a healing agent—was studied. A repeat test was carried out after 14 days at 20, 40, or 60 °C. The results of determining the tensile strength during splitting are presented in Figure 8.

Analysis of Figure 8 shows that insignificant strength is observed during the splitting of SMA samples without the use of a reducing agent. In this case, an increase in the temperature of the environment in which the reduction occurs leads to an increase in the tensile strength during repeated testing, which is consistent with the previously obtained results [47,48,49]. Thus, when the temperature increases to a temperature close to the softening temperature of bitumen, an increase in strength is observed after self-healing. This effect is due to a change in the rheological properties of the matrix, a decrease in the viscosity of bitumen, and an increase in the mobility of the molecules. In this state, the probability of spontaneous entanglement of molecules among themselves and their fusion through the restoration of specific bonds increases [50,51]. Moreover, the intensity of this process depends on the intermolecular distance and the speed of thermal movement of the molecules, which naturally increases with increasing temperature. In this case, in the area of the defect (crack), if its surfaces are sufficiently close, contact may occur, facilitating their spontaneous fusion.

The presence of sunflower oil between the defect planes in SMA samples contributes to an increase in the re-determined strength by 2.5 times in comparison with the traditional SMA samples. And with increasing temperature, this figure increases 6 times. This is due to an additional increase in the mobility of bitumen molecules dissolved in the additional volume of the oil medium due to the healing agent, as well as the presence of drying oils in the composition of sunflower oil, which tend to polymerize when the temperature rises. This is consistent with the results presented in [52,53,54].

When gluing samples with a polymer healing agent, the splitting strength is 0.11–0.82 MPa after a rest period in an environment with a temperature of 20 to 60 °C, respectively. The mechanism of action of ARP differs from SfO, since it provides strength restoration due to the formation of adhesive bonds as a result of polymerization. An increased temperature increases the intensity of this process and the strength of the bonds formed.

Thus, two different healing agents, SfO and ARP, having a different mechanism of action, are able to restore the mechanical characteristics of asphalt concrete when the temperature of the environment in which asphalt concrete is restored increases. Greater strength is observed at a temperature close to the softening temperature of the bitumen used. However, under such conditions, asphalt concrete loses its load-bearing capacity, is prone to excessive deformation, and cannot be used, so using the ability of bitumen molecules to spontaneously coalesce as a mechanism for extending the serviceability of coatings is difficult.

A promising option appears to be a polymer healing agent. The effectiveness of recovery due to the agent can then be regulated by adding activators that accelerate the process of its polymerization.

This work studied the effect of an activator consisting of a mixture of sulfur, manganese (IV) oxide, and tetramethylthiuram disulfide in a ratio of 6.1:3.7:1.0. To do this, when manufacturing half-cylinder samples of SMA, an activator was added to the mixture in an amount of 0.55–11.3% by weight of bitumen. Bonding of samples using ARP after splitting was carried out using the technology described earlier. The ambient temperature during the rest period was 20 °C. The assessment of the influence of the activator content in the composition of SMA on the ARP deposition potential was carried out based on the relative strength:

(13) $$k_{R}=\frac{R_{i}}{R_{0}}\cdot 100\%,$$

where *R*_0_ and *R_i_* are the ultimate splitting strength of SMA-15 samples before and after gluing, respectively.

**Figure 9:**
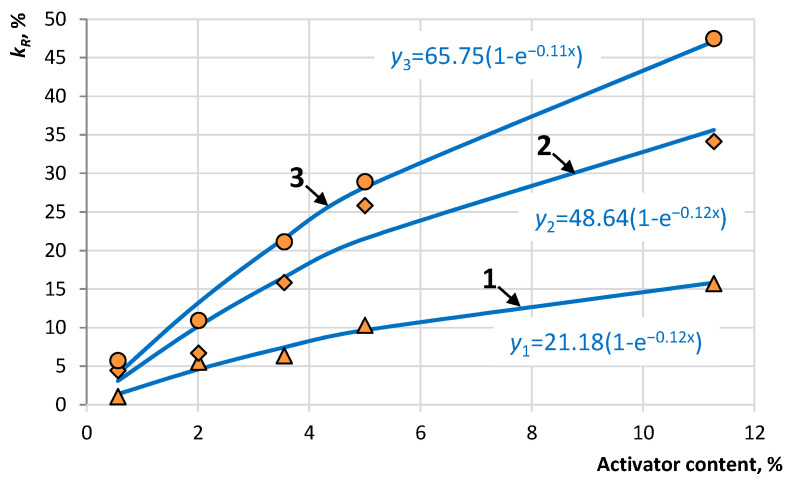
Dependence of the relative strength on the activator content after a recovery period: 1—3 days, 2—7 days, and 3—14 days.

The results of determining the change in relative strength from the concentration of the activator in the composition of the crushed stone-mastic asphalt concrete mixture are presented in Figure 9.

**Figure 10:**
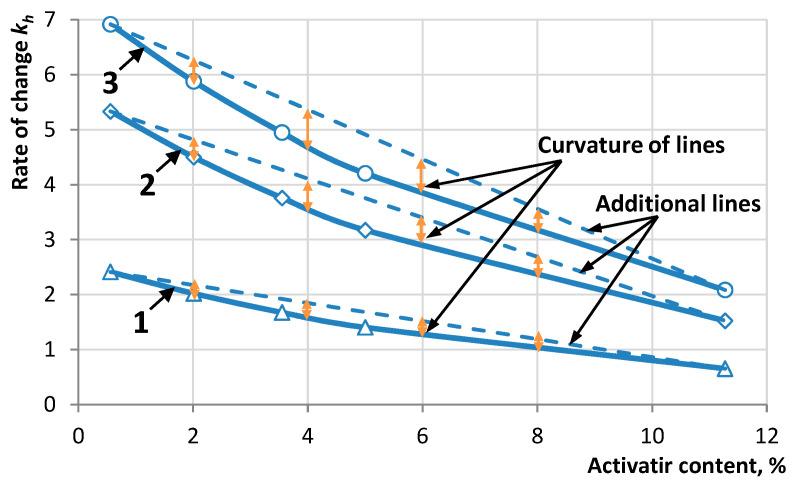
Dependence of the rate of relative strength on the activator content after a recovery period: 1—3 days, 2—7 days, and 3—14 days.

The data in Figure 8 show that the use of an activator makes it possible to obtain repeated strength when splitting SMA samples after gluing ARP, the values of which range from 5 to 45% of the original. The change in relative strength is described by the exponential dependence *y* = *y*_max_(1 − e^−*bx*^), for which the rate of strength increase is not constant (Figure 10).

**Figure 11:**
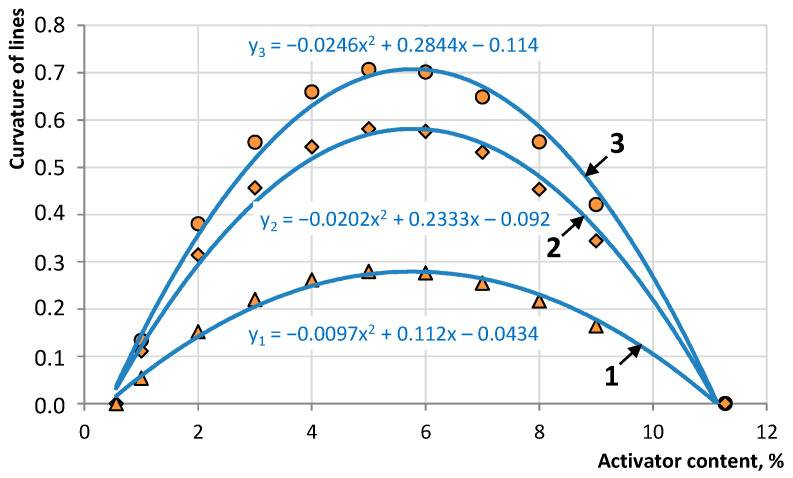
Dependence of the curvature of the lines on the activator content after a recovery period: 1—3 days, 2—7 days, and 3—14 days.

The graph of the rate of change in relative strength can be characterized by two stages: the initial one with a high rate of change and the final one with a slowdown in the rate, which tends to minimum values. On the graphs, the characteristic points of transition from the first stage to the second are the points that were determined taking into account the radius of curvature of the curve. The curvature of the lines *k_h_*(*c*) was estimated mathematically by the magnitude of deviations of the curve from the auxiliary line *f*(*c*), where the maximum difference in the values of *k_h_* from *f*(*c*) corresponds to the inflection point of the dependence under study. The results of determining the described deviation value are presented in Figure 11.

The dependence of the curvature of the curves under study is described by a quadratic function, which makes it possible to determine the extremum values using the first derivative, which will correspond to the inflection point. The maximum curvature value for the function describing the rate of change in the relative strength of the samples after a recovery period of 3, 7, and 14 days corresponds to an activator concentration of 6.2%, 5.8%, and 5.8%, respectively. When the activator concentration increases to more than 5.9% of the mass of bitumen in the asphalt concrete mixture, the efficiency of a unit of its volume decreases. To use an activator at a higher concentration, it is necessary to conduct an economic feasibility study.

In order to systematize research in the field of self-healing asphalt concrete and expand the list of healing agents that can ensure the formation of a healing effect, an approach has been proposed to assess the suitability for encapsulation using alginate emulsions.

Traditionally, the choice of a healing agent in capsules for asphalt concrete is determined by its ability to rejuvenate bitumen, and therefore, the main components for encapsulation are hydrocarbon compounds [55,56] or industrial rejuvenators [57,58]. Given their similar nature to bitumen [59] and based on experience with their use, they are potentially suitable for use as a healing agent. The degree of their effectiveness will depend both on the group composition and on the presence of additional components, such as polymers. For an objective choice in favor of the preferred option, a quantitative assessment of the degree of affinity of a substance with bitumen using the solubility index and/or Gibbs energy of a binary system can be used. The difference in the solubility coefficient of bitumen and SfO is 1.2 (J/cm^3^)^0.5^, and for ARP Δδ = −0.6 (J/cm^3^)^0.5^, which indicates its better solubility and ability to form a homogeneous system. This was confirmed by the Gibbs energy values for binary systems, which have a value ∆*G* < 0, and the “AR-polymer–bitumen” system has the highest absolute values.

When using sodium alginate for the synthesis of capsules with healing agents, an important condition for the implementation of the technology is the possibility of obtaining emulsions. This condition can be verified qualitatively using an optical microscope or quantitatively using the dynamic viscosity index at a constant or variable shear rate [46,60]. In this case, the presence of a dispersed phase in the form of drops of a healing agent will contribute to the manifestation of the properties of a non-Newtonian liquid.

Given the different mechanisms of action of healing agents, the choice must be made taking into account the potential that they can provide. In this work, it is proposed to assess the change in tensile strength upon splitting of samples glued together with a healing agent. The strength of the samples that were restored without a filling agent was 3.7% of the initial value. In the case of sunflower oil, the effectiveness of gluing will depend on the degree of matrix rejuvenation at the crack shores, which ensures spontaneous entanglement of hydrocarbon molecules with each other [49,53,61]. Under the influence of SfO, the splitting strength was 14.2% of the initial value. For a polymer healing agent, the restorative effect is due to the formation of new bonds during polymerization, which will depend on the intensity of this process. Under the influence of ARP, the splitting strength was 5.8% of the initial value. At the same time, the proposed method allows one to evaluate the intensity of the polymerization process of the healing agent in the presence of activators located in the asphalt concrete matrix. This allows you to increase the restorative effect to 25–45% of the initial strength.

Thus, the developed approach allows us to evaluate the suitability of a substance for use as a healing agent when encapsulated using alginate technology and make a choice in favor of more effective options. The proposed methods are universal and can be used to evaluate various types of healing agents working as rejuvenators or adhesives. Technological criteria (solubility index, Gibbs energy, and rheological properties) will exclude various therapeutic agents that are not suitable for the synthesis of alginate capsules. Physic-mechanical criteria (adhesive strength) will allow you to select a healing agent with the best healing effect. The use of such methods will make it possible to systematize research in the field of development of self-healing asphalt concrete, as well as significantly expand the list of healing agents potentially suitable for encapsulation and ensuring the formation of a healing effect.

## 4. Conclusions

An approach to studying a substance is proposed to assess the suitability of its use as a healing agent during encapsulation using alginate technology in terms of solubility, homogeneity in a system with bitumen, and adhesive strength. This set of indicators can be used in the development and design of self-healing asphalt concrete, as well as to expand the list of healing agents that can be used to implement self-healing technology.

The results of this work prove that substances with different mechanisms of action can be used as healing agents for the synthesis of capsules when implementing self-healing technology in asphalt concrete. Among such substances, rejuvenators or polymers can be considered as filling agents. It has been proven that the intensity of the self-healing process using polymer healing can be increased through the use of activators in the composition of asphalt concrete mixtures. An example of such a healing agent is thiol-containing urethane polymers with terminal mercaptan groups (SH-)—AR-polymers, the activator for which can be a mixture of technical sulfur, manganese oxide (IV), and tetramethyl thiuram disulfide at a ratio 6.1:3.7:1.0.

## Figures and Tables

**Table 1:** Methods for determining solubility.

Formula	Number	Notes	References
$\delta_{i}=\sqrt{\frac{∆H_{v}-RT}{V_{m}}}$	(5)	Δ*H_v_* is the heat of evaporation of a substance; *R* is the gas constant; *T* is the absolute temperature; *V_m_* is the molar volume	[25]
$\delta_{i}=4.1{\left(\frac{\sigma_{i}}{\sqrt[{3}]{v_{i}}}\right)}^{0.43}$	(6)	*v_i_* is the molar volume of the substance; *σ_i_* is the surface tension of the substance	[26]
$\delta_{i}=\frac{\gamma \sum G}{M}$	(7)	γ is the density of matter; *M* is the molecular weight of the substance (unitary polymer unit); Σ*G* is the sum of the attraction constants of individual atomic groups of a substance (an elementary unit of a polymer)	[27]

**Table 2:** Properties of ARP.

Parameter	Unit	Value
Dynamic viscosity at 25 °C	Pa·s	9.7
SH-group content	%	1.5–2.5
Tensile strength after curing	MPa	1.0
Elongation at break	%	100
Permanent deformations	%	until 6

**Table 3:** Properties of SfO.

Parameter	Unit	Value	Method
Viscosity at 25 °C	Pa·s	0.05	–
Density at 25 °C	g/cm^3^	0.918 ± 0.05	[29] [30]
Acid value	mg KOH/g	0.025 ± 0.01	[31]
Fractional composition:			
palmitic acid	%	6.61	[32]
stearic acid	%	3.61	
oleic acid	%	30.91	
linoleic acid	%	57.13	
other	%	1.74	

**Table 4:** Sieve grain analysis (particle size distribution).

Parameter	Value								
Sieve size, mm	15	10	5	2.5	1.25	0.63	0.315	0.16	0.071
Passing, %	92.3	58.8	33.0	21.7	18.4	16.5	14.7	12.7	10.6

**Table 5:** Properties of bitumen.

Parameters	Unit	Value	Method
Penetration at temperature 25 °C	0.1 mm	67	[35] [36]
Penetration at temperature 0 °C	0.1 mm	36	
Ductility at temperature 25 °C	mm	85.2	[37] [38]
Ductility at temperature 0 °C	mm	3.6	
Softening point	°C	51	[39] [40]
Fraass breaking point	°C	−20	[41]

**Table 6:** Properties of components and their ability to form a homogeneous mixture.

Component	ρ, g/cm^3^	*M*, g/mol	δ, (J/cm^3^)^0.5^	Δδ, (J/cm^3^)^0.5^
Bitumen	0.986	1050	2.7	–
AR-polymer	1.070	3200–3400	2.1	−0.6
Sunflower oil	0.918	879	3.9	1.2

**Table 7:** The main properties of SMA-15.

Parameter	Unit	Standard Limits	Value
Density	g/cm^3^	–	2.43
Mineral framework porosity	%	15–19	18
Air voids content	%	1.5–4.5	3.0
Water saturation	%	1.0–4.0	1.9
Compressive strength at 20 °C	MPa	at least 2.2	3.0
Compressive strength at 50 °C	MPa	at least 0.65	1.10
Shear resistance: internal friction coefficient	–	at least 0.93	0.94
Shear resistance: shear grip at 50 °C	MPa	at least 0.18	0.56
Breaking tensile strength at 0 °C	MPa	2.5–6.0	2.6
Splitting strength at −20 °C	MPa	–	1.9
Water resistance	–	at least 0.85	0.92

**Figure A1:**
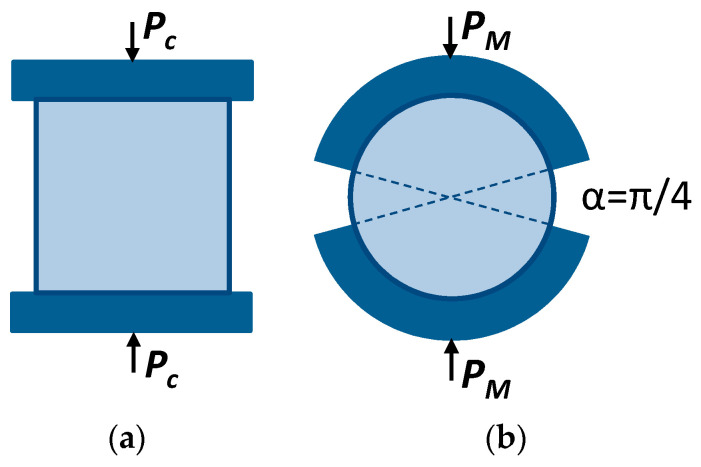
Scheme of the stress-strain states under uniaxial compression (**a**) and compression according to the Marshall scheme (**b**).

## Data Availability

Data are contained within the article.

## Appendix A

The main properties of SMA-15 were determined according to the methods of State standard GOST 12801-98.

Determination of the average density: the essence of the method is to determine by hydrostatic weighing the average density of samples made in the laboratory or selected from the structural layers of road pavements, taking into account the pores present in them. The average density of a sample from a mixture *ρ*m, g/cm^3^ is calculated using the formula:

(A1) $$\rho_{m}=\frac{m\rho_{w}}{m_{2}-m_{1}},$$

where *m* is the mass of the sample suspended in air; ρ*_w_* is the density of water equal to 1 g/cm^3^; *m*_1_ is the mass of the sample suspended in water; and *m*_2_ is the mass of the sample kept for 30 min in water and then re-weighed in air (all masses expressed in grams).

Determination of the mineral framework porosity: the essence of this method is to determine the volume of pores present in the mineral part (framework) of the compacted mixture or asphalt concrete. Mineral framework porosity *V_m,p_*, %, is calculated to the first decimal place using the formula:

(A2) $$V_{m,p}=\left(1-\frac{\rho_{m,f}}{\rho_{f}}\right)\cdot 100,$$

where ρ*_m_*_,*f*_ is the average density of the mineral part of asphalt concrete, g/cm^3^; and ρ*_f_* is the true density of the mineral part of the mixture, g/cm^3^.

Determination of air voids content: air voids *V_a_*, %, is determined by a calculation based on the pre-established average and true densities and is accurate to the first decimal place:

(A3) $$V_{a}=\left(1-\frac{\rho_{m}}{\rho }\right)\cdot 100,$$

where ρ is the true density of the asphalt concrete mixture, g/cm^3^.

Determination of water saturation: the essence of this method is to determine the amount of water absorbed by the sample at a given saturation mode. The vessel with the samples is placed under vacuum, where a pressure of no more than 2000 Pa (15 mm Hg) is created and maintained for 1 h. Then, the pressure is brought to atmospheric pressure and the samples are kept in the same vessel with water at a temperature of 20 ± 2 °C for 30 min. After this, the samples are removed from the vessel, wiped with a soft cloth, and weighed in air. Sample water saturation W, %, is calculated by the formula:

(A4) $$W=\frac{m_{3}-m}{m_{2}-m_{1}}\cdot 100,$$

where *m*_3_ is the mass in grams of a water-saturated sample suspended in air.

Determination of the compression test: the essence of this method is to determine the load required to destroy the sample under the given conditions. Before testing, the samples are brought to a certain temperature: 50 ± 2 °C, 20 ± 2 °C. Next, the sample is placed in the center of the lower plate of the press, then the electric motor of the press is turned on and the sample begins to be loaded at a press plate movement speed of 3.0 ± 0.3 mm/min. The maximum reading of the force meter is taken as the breaking load. Ultimate compressive strength *R_c_*, MPa, is calculated using the formula:

(A5) $$R_{c}=\frac{P}{F}\cdot 10^{-2},$$

where *P* is the breaking load in N; *F* is the initial cross-sectional area of the sample in cm^2^; and 10^−2^ is the conversion factor to MPa.

Determination of the breaking tensile strength: the essence of this method is to determine the load required to split the sample along the generatrix. Before testing, the samples are brought to 0 ± 2 °C for at least 1 h in water. Then, the sample is placed in the center of the bottom plate of the press on the side surface, the electric motor of the press is turned on, and the sample begins to be loaded at a press plate movement speed of 50 ± 1 mm/min. The maximum reading of the force meter is taken as the breaking load. The ultimate tensile strength at splitting *R_s_*, MPa, is calculated using the formula:

(A6) $$R_{s}=\frac{P}{hd}\cdot 10^{-2},$$

where *P* is the breaking load in N; *h* is the sample height in cm; *d* is the sample diameter in cm; and 10^−2^ is the conversion factor to MPa.

Determination of shear resistance: the essence of this method is to determine the maximum loads and corresponding limiting deformations of standard cylindrical samples under two stress-strain states (Figure A1): under uniaxial compression and under compression according to the Marshall scheme.

Before testing, the samples and crimping device are kept for 1 h at 50 ± 2 °C in water. Half of the samples are tested according to the first loading scheme, the other half according to the second. The sample is installed in the center of the lower plate of the press for the first compression scheme or in the lower part of the crimping device for the second compression scheme, the press electric motor is turned on, and the sample begins to be loaded at a press plate movement speed of 50 ± 1 mm/min. During the test, the maximum reading of the force meter and the maximum deformation corresponding to the breaking load are recorded. For each sample, the work *A*, J, spent on destruction is calculated using the formula:

(A7) $$A=\frac{Pl}{2},$$

where *P* is the breaking load in kN; and *l* is the ultimate deformation in mm.

The average work of deformation of the samples under uniaxial compression and compression according to the Marshall scheme is calculated as the arithmetic mean value of the test results of at least three samples. The coefficient of the internal friction of the asphalt concrete tgϕ and shear adhesion *S_g_*, MPa, are calculated using the formulas:

(A8) $$tg\varphi =\frac{3\left(A_{m}-A_{c}\right)}{3A_{m}-2A_{c}},$$

where *A_m_* and *A_c_* are the average work of deformation of asphalt concrete samples when tested according to the Marshall scheme and under uniaxial compression, respectively, in J, and:

(A9) $$S_{g}=\frac{1}{3}\left(3-2tg\varphi \right)R_{c},$$

where *R_c_* is the ultimate strength in uniaxial compression, determined by (A5) in MPa.

Determination of the shear resistance: the essence of this method is to assess the degree of loss of compressive strength of samples after exposure to water under vacuum conditions. Water resistance *k_w_* is calculated to the second decimal place using the formula:

(A10) $$k_{w}=\frac{R_{c,w}}{R_{c}}$$

where *R_c_*_,*w*_ and *R_c_* are the ultimate strength under uniaxial compression of a sample saturated with water in a vacuum and without water saturation, respectively, determined by (A5), in MPa.
